# Fabrication of Mesoscale Channel by Scanning Micro Electrochemical Flow Cell (SMEFC)

**DOI:** 10.3390/mi8050143

**Published:** 2017-05-04

**Authors:** Cheng Guo, Jun Qian, Dominiek Reynaerts

**Affiliations:** Department of Mechanical Engineering, KU Leuven & Member Flanders Make, Leuven 3001, Belgium; cheng.guo@kuleuven.be (C.G.); dominiek.reynaerts@kuleuven.be (D.R.)

**Keywords:** electrochemical machining (ECM), scanning micro electrochemical flow cell (SMEFC), micro-ECM, channel machining

## Abstract

A unique micro electrochemical machining (ECM) method based on a scanning micro electrochemical flow cell (SMEFC), in which the electrolyte is confined beneath the tool electrode instead of spreading on the workpiece surface, has been developed and its feasibility for fabricating mesoscale channels has been investigated. The effects of the surface conditions, the applied current, the feed rate, the concentration of the electrolyte and several geometrical parameters on the machining performance have been investigated through a series of experiments. The cross-sectional profile of the channels, the roughness of the channel bottom, the width and depth of the channel, the microstructures on the machined surface and the morphologies of the moving droplet have been analyzed and compared under different machining conditions. Furthermore, experiments with different overlaps of the electrolyte droplet traces have also been conducted, in which the SMEFC acts as a “milling tool”. The influences of the electrode offset distance (EOD), the current and the feed rate on the machining performance have also been examined through the comparison of the corresponding cross-sectional profiles and microstructures. The results indicate that, in addition to machining individual channels, the SMEFC system is also capable of generating shallow cavities with a suitable superimposed motion of the tool electrode.

## 1. Introduction

Nowadays, there is an increasing demand of mesoscale channel structures in many industrial domains such as fuel cells [1], hydrodynamics bearing [2], and sealing ring channel [3]. Electrochemical machining (ECM) has proven to be a unique method for fabricating channels, ranging from the micro scale to the macro scale, with excellent surface integrities on metallic materials. Ryu [4] utilized a micro foil electrode instead of a micro-shaft electrode to achieve micro grooving in the environment of the citric acid electrolyte and a 42 µm wide and 18 µm deep single channel with 11 nm Ra surface roughness was obtained. Liu et al. [5] has utilized a kind of ECM method with low-frequency vibrations to fabricate multiple slots for the application of fuel cells. The same features were also successfully fabricated by other researchers [6], in which they developed an innovative multifunctional cathode, combining the tool electrode, the sealing device and the spacers between every channels. Electrolyte jet machining (jet-ECM) is also a promising technique for channel machining. Natsu et al. [7] machined complex channels on a thrust hydrodynamic bearing surface with jet-ECM in an efficient way. Compressed air assisted jet-ECM was also attempted to fabricate channels on the workpiece made of Nimonic Alloy 80a [8]. Instead of using conventional jet-ECM with a round nozzle scanning on the workpiece to fabricate channels [9], Kunieda et al. [10] utilized a flat nozzle to shorten the channel machining time. With the same method, Hackert-Oschätzchen et al. [11] fabricated complex channels with a width below 200 µm for the application of microfluidics and micro reactors. Apart from these methods, electrochemical milling has also been verified as a feasible method for channel machining. As an example, Ghoshal and Bhattacharyya [12] used micro tools with different front end shape for microchannel machining with the scanning machining layer and layer method. Furthermore, they investigated the optimum scan feed rate in electrochemical micromachining of micro channels and found that the introduction of the optimum scan feed rate can reduce overcut and avoid the breakage of micro tools during the grooving process [13]. Besides, Kim et al. [14] utilized the electrode with a diameter of 38 µm to fabricate a 50 µm wide micro channel thanks to ultrashort pulses. Zhang et al. [15] also used an electrode with the diameter of 10 µm to machine micro channels with the width of 20 µm. For machining micro through-grooves, the micro wire electrode electrochemical cutting method is also a promising way. Shin et al. [16] used a 10 µm diameter tungsten wire as the tool electrode and obtained micro channel of around 20 µm in width on stainless steel. Wang et al. [17] obtained multi-microchannels by applying low frequency and small amplitude vibration on the wire electrode based on the conventional setup. Recently, hybrid processes such as ECM with slurry jet [18], laser-assisted jet-ECM [19] and laser-assisted ECM milling [20] have brought in new possibilities for the efficient fabrication of channels on hard metals with enhanced machining localization.

Currently, the growing environment awareness in the research community has brought forth a trend for clean manufacturing in the domain of electro-physical and chemical machining. Ryu [4] has proposed a micro electrochemical reverse drilling method, in which the electrochemical reactions are confined in a droplet formed at the bottom edge of the workpiece. The droplet can be stabilized by the surface tension and the gravity, keeping other regions untouched. Sakairi et al. have developed a co-axial dual capillary solution flow type droplet cell to accomplish Ni deposition [21]. Unlike in the traditional case, the deposition happens in a container filled with electrolyte. Drensler et al. [22] proposed a method to use scanning droplet cell to selectively dissolve NiAl matrix and release the embedded W nanowires, which is intended for the application of self-assembly. Hu et al. [23] also proposed a gushing and sucking method with a coaxial tube to achieve the groove width of 103 µm and the surface roughness of 0.012–0.025 µm. Kuo et al. [24] tried to process quartz glass by electrochemical discharge machining (ECDM) with titrated flow of electrolyte, leading to less cost and pollution because of the electrolyte supplied in droplets. These methods have some special advantages compared with the conventional ones, such as keeping non-processing region untouched, better safety for operators and more feasibility to be integrated into other process chains.

In this research work, a scanning micro electrochemical flow cell (SMEFC) has been proposed to generate channels on metallic workpieces. In the SMEFC, the electrolyte is confined in a small droplet and its refreshing is simultaneously maintained. In this way, electrolyte splashing does not exist, so there is no need of an electrolyte tank for the machining region. As a result, this technique can be conveniently integrated into other manufacturing process because of its unique control of the electrolyte. The influence of surface condition on the machining performance was investigated firstly. Then, several process parameters (e.g., the current, the feed rate and the concentration of the electrolyte) and geometrical parameters have also been varied to investigate the effects on the channel formation process, in terms of dimensional parameters and surface microstructures. After analyzing the machining of single channels by SMEFC, superimposed process of SMEFC with different lateral overlapping has also been examined to study the feasibility of SMEFC for electrochemical milling.

## 2. SMEFC Experimental System

**Figure 1:**
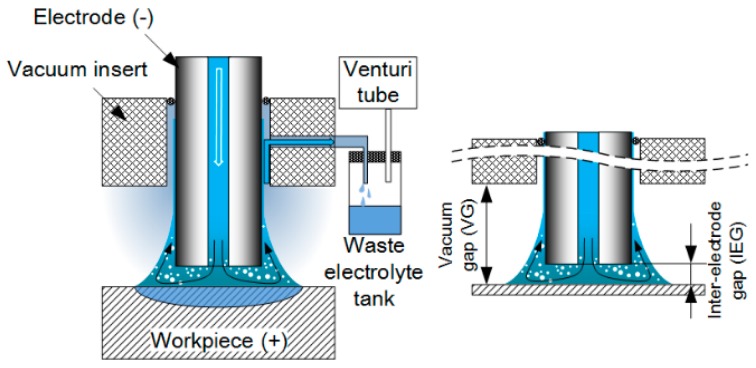
Schematics of the scanning micro electrochemical flow cell (SMEFC).

Figure 1 shows the schematics of the SMEFC system. The principle of the electrolyte circulation is that the electrolyte is pumped through a hollow electrode and then it arises along the electrode outer wall by the surrounding flowing air induced by the Venturi effect, resulting in a droplet between the electrode and the workpiece. This method maintains the electrolyte of the droplet fresh and confines the electrolyte in the region of interest. The used electrolyte with the reaction products flows eventually into the waste electrolyte tank through a channel in the suction head. The vacuum gap (VG) and the inter-electrode gap (IEG) can be adjusted.

**Figure 2:**
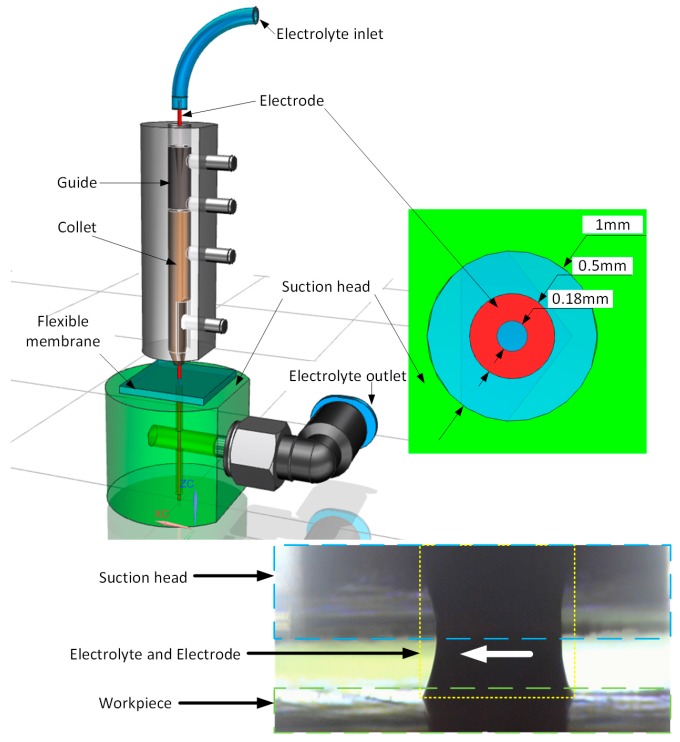
Layout of SMEFC setup.

The solid model of the experimental setup in Figure 2 depicts the tool electrode is positioned in the collet. The suction head can move up and down through manually tuning a stage. The stage can also adjust the hole of the suction head to the center of the electrode. A flexible membrane is used to seal the suction head for the recycling of the electrolyte. The hole diameter in the suction head is 1 mm. The electrode is made of tungsten carbide, with an outer diameter of 0.5 mm and an inner diameter of 0.18 mm. The electrode is glued with a flexible tube, through which the electrolyte is pumped, as shown in Figure 2. The actual layout of the suction head, workpiece and the electrode wrapped with electrolyte are shown in Figure 2.

The ECM power supply is a homemade switching power generator, working at 150 kHz. This generator can be set to either constant-current or constant-voltage working mode. The output ripple is less than 20 mV and the response time is less than 50 ms. The motorized stage is MTS25-Z8 of THORLABS with the travel range of 25 mm and the maximum velocity of 2.4 mm/s. The minimum achievable incremental movement is 0.05 µm and the bidirectional repeatability is 1.6 µm.

The cross-sectional profiles and the roughness of the channels machined by SMEFC have been measured by a Mitutoyo CS3200 profiler (Mitutoyo, Kawasaki, Japan). When measuring the roughness, the workpiece is cleaned firstly in a mixture of ethanol and acetone in an ultrasonic tank. The stylus contacts the bottom surface of the channel and moves along the channel. The nominal radius of the stylus is 2.0 µm. A Zeiss optical microscope (Carl Zeiss AG, Oberkochen, Germany), SteREO Discovery V20, was used to evaluate the width of the channels cavities, and Dino-Lite digital microscope AM4115ZT (AnMo Electronics Corporation, Hsinchu, Taiwan) was installed to help monitor and observe the moving electrolyte droplet above the workpiece top surface. The maximum lateral resolution of the digital microscope is around 1.4 µm/pixel. The surface microstructures of the channel bottom surface were examined with a Phenom desktop SEM (Phenom-World B.V., Eindhoven, The Netherlands). The current signals during the electrochemical dissolution were recorded by a data acquisition unit embedded in the ECM power supply.

## 3. SMEFC Machining Experiments and Discussion

A series of experiments have been carried out under constant-current mode. The electrolyte is a sodium nitrate (NaNO_3_) solution with the concentration of 120 g/L and 250 g/L. The workpiece material is a kind of stainless steel from UDDEHOLM STAVAX^®^ ESR (Hagfors, Sweden). The electrolyte flow rate is 0.06 mL/s pumped by a metering pump of ProMinent^®^ (ProMinent GmbH, Heidelberg, Germany). The vacuum condition is achieved by a Venturi tube, with an inlet pressure of 4 bar. The corresponding vacuum pressure is 45 kPa.

### 3.1. Experimental Verification

When the droplet moves on the workpiece, the droplet shape will be regulated by its gravity, surface tension and adhesion simultaneously. Therefore, surface conditions or the roughness of the workpiece may affect the behavior of the moving droplet. To investigate this conjecture, samples with two different surface treatments were used. One sample was ground by a surface grinder (*R*a = 0.4 µm) and the other one was pre-machined by micro-EDM milling (*R*a = 1.2 µm). The surface tension of the electrolyte is around 75.5 mN/m [25].

**Figure 3:**
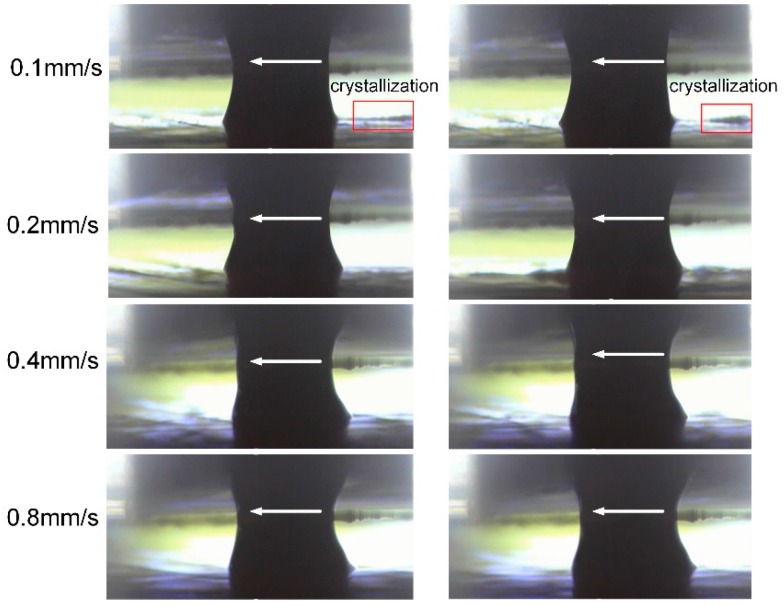
Electrolyte droplet moving on the surface treated by micro-electrical-discharge machining (EDM) milling.

**Figure 4:**
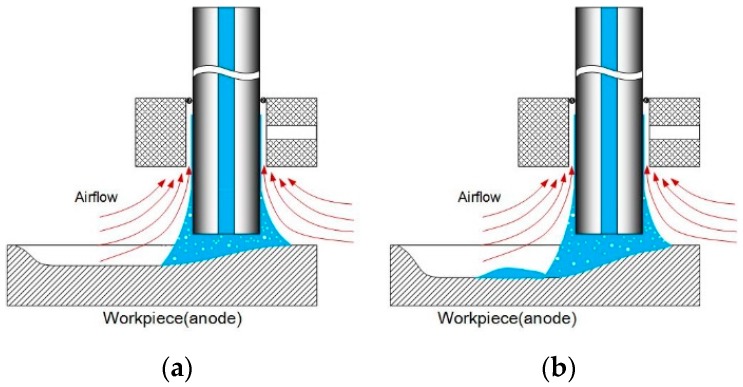
Explanation of the electrolyte leakage. (**a**) Surface tension maintains a single droplet; (**b**) electrolyte left on machined surface once the droplet volume is larger.

Figure 3 shows the profile of the electrolyte droplet moving at different speed on a micro-EDMed surface. The moving direction of the electrolyte droplet relative to the workpiece has been indicated by the arrows. The applied current is 230 mA and the feed rates are 0.1 mm/s, 0.2 mm/s, 0.4 mm/s and 0.8 mm/s, respectively. It can be noticed that when the scanning speed is 0.1 mm/s, the droplet seems to be not stable. The electrolyte left on the workpiece can be obviously observed and it crystallizes very quickly probably due to its exposure to surrounding flowing air. With a slower feed rate, the channel becomes deeper and the corresponding droplet volume in the channel becomes bigger in the same time. When the volume reaches a certain level, the surface tension (cohesion) cannot support the volume in the form of a single droplet. As a result, some of the electrolyte is left on the machined surface, as illustrated in Figure 4. Consequently, the electrolyte cannot be fully recovered by the Venturi effect. The electrolyte droplets above the workpiece top surface under the other three feed rates maintain a relatively stable shape during the motion. However, when the feed rate goes even higher, the cross-sectional profile perpendicular to the moving direction becomes increasingly asymmetrical.

**Figure 5:**
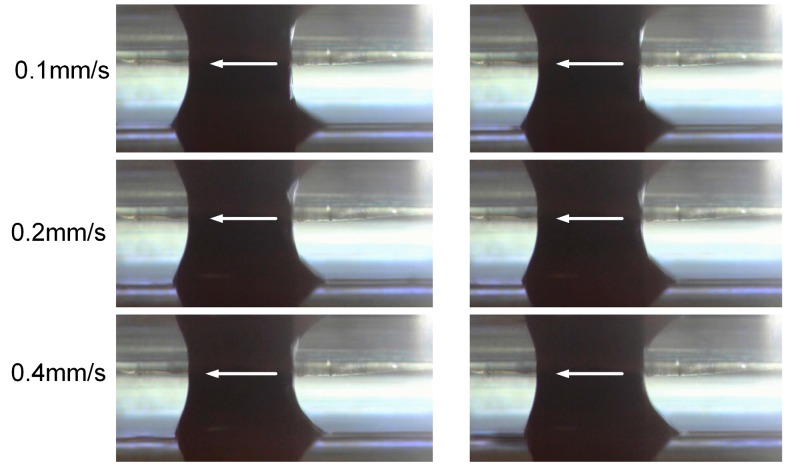
Electrolyte droplet moving on the ground surface.

Figure 5 depicts the situation when the electrolyte droplet moves on the ground surface with the feed rates of 0.1 mm/s, 0.2 mm/s and 0.4 mm/s, respectively. Obvious delaying of the electrolyte droplet can be noticed. Compared with the case in Figure 3, the trail above the workpiece top surface becomes longer. Obviously, this difference in the droplet morphology is induced by the difference in the roughness of these two samples.

Comparing the droplet profiles in Figure 4 and Figure 5, it can be concluded that the surface roughness indeed influences the morphology of the moving droplet and further affects the flow field and the electric field distribution. After this initial stage of comparison, only ground samples were used in the further experiments.

### 3.2. Effects of Current Density and Feed Rate

**Figure 6:**
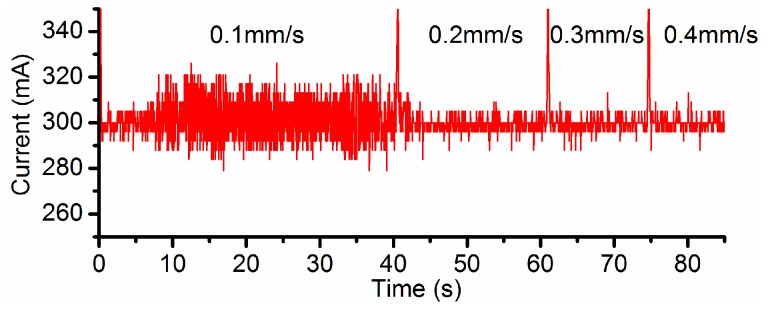
Current signal when setting 300 mA as the target.

Different current settings, i.e., 100 mA, 200 mA and 300 mA, have been, respectively, applied in the channel machining under a variety of feed rate of 0.1 mm/s, 0.2 mm/s, 0.3 mm/s and 0.4 mm/s. The vacuum gap (VG) and the inter-electrode gap (IEG) were set to 200 µm and 50 µm respectively. When the feed rate is set to 0.1 mm/s, relatively large fluctuations in the current signals take place around 300 mA (Figure 6), in comparison to the cases under the other three feed rates. It also means the flow field under this feed rate is unstable. Therefore, the electricity consumption per unit length (ECPL) is defined in this research and its value should be limited in order to obtain a stable flow field and to avoid low field instability.

In order to evaluate the effects of the feed rate and the current on the grooving performance, the ECPL was first set at a constant value in the experiments. Then, the values of current and the feed rate were varied within a range according to this rule. The first three columns of Table 1 list the five groups of parameters utilized in the experiments, with the ECPL being 1 C/mm and 0.5 C/mm, respectively.

The experimental results were compared with the theoretical results derived by Faraday’s law. Faraday’s law can be summarized as,

(1) $$m_{the}=\frac{M\times Q}{F\times n}$$

where *m_the_* is the mass of the substance, *Q* the total electric charge passed through the substance, *F* = 96,485 C/mol the Faraday constant, *M* the molar mass of the substance and *n* the valency number of the ions of the substance.

After substituting *Q* with ECPL, the theoretical cross-sectional removal area *S_the_* by SMEFC can be written as,

(2) $$S_{the}=\frac{M\times \mathrm{ECPL}}{F\times n\times \rho }$$

where *S_the_* is the theoretical cross-sectional area and ρ is the density of the substance.

The current efficiency can be described as,

(3) $$\eta =\frac{m_{exp}}{m_{the}}=\frac{S_{exp}}{S_{the}}$$

where *m_exp_* and *S_exp_* are the experimental removal mass and experimental cross-sectional area, respectively. The valency number *n* of stainless steel is controversial, because it depends on the proportion of the generated Fe^2+^ ions and Fe^3+^ ions. According to the results of [26], when Fe^2+^:Fe^3+^ = 1:2, *n*_1_ = 3.1410. Using this value, the current efficiency η is calculated to be always larger than 100%. Similar results are also shown in [26], and the uncertain proportion of Fe^2+^ ions and Fe^3+^ ions induce this phenomenon. Therefore, *n*_2_ = 2.5786 is also used in the calculations, which is obtained under the assumption that only Fe^2+^ ions are generated. η_1_ and η_2_ represent the current efficiency calculated under *n*_1_ and *n*_2_ respectively. The actual current efficiency should be a value between these two efficiencies. *S_the_* is calculated under the valency number of *n*_2_.

**Figure 7:**
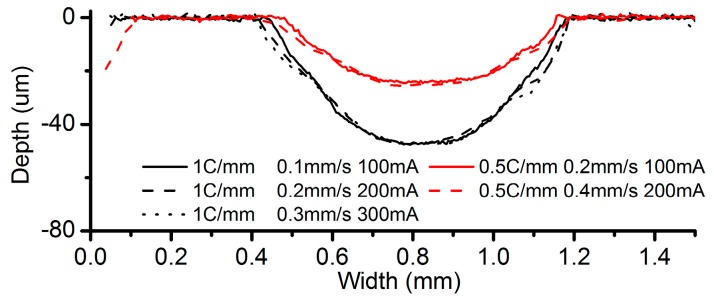
Cross-sectional profiles.

**Figure 8:**
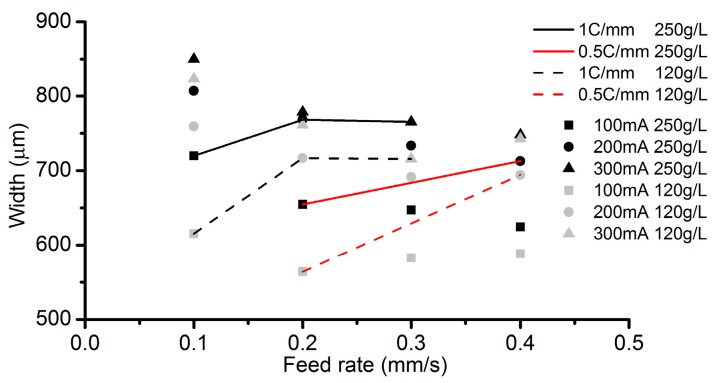
Channel width comparison.

Figure 7 shows the cross-sectional profiles of the channels machined with the parameters in Table 1. It can be observed that when the same ECPL, the maximum depth of these channels is almost the same in spite of some slight differences (Figure 7). However, as shown in Figure 8, the width of the channels has clear deviations. At the ECPL of 1 C/mm, the channel width processed by 0.1 mm/s is the smallest and almost the same width is produced in the cases of 0.2 mm/s and 0.3 mm/s. Similarly, at the ECPL of 0.5 C/mm, the channel width increases with the feed rate. A high concentration (250 g/L) alleviates the trend. There are two possible reasons accountable for this trend. One reason is that a high current can derive a high current efficiency compared to a low current, which can be confirmed in the current efficiency in Table 1. The other possible reason is that the contact angle of the electrolyte droplet is also influenced by the applied voltage. This implies that if a relative voltage is applied, the electrolyte droplet needs to expand to a bigger area to obtain a smaller contact angle.

**Figure 9:**
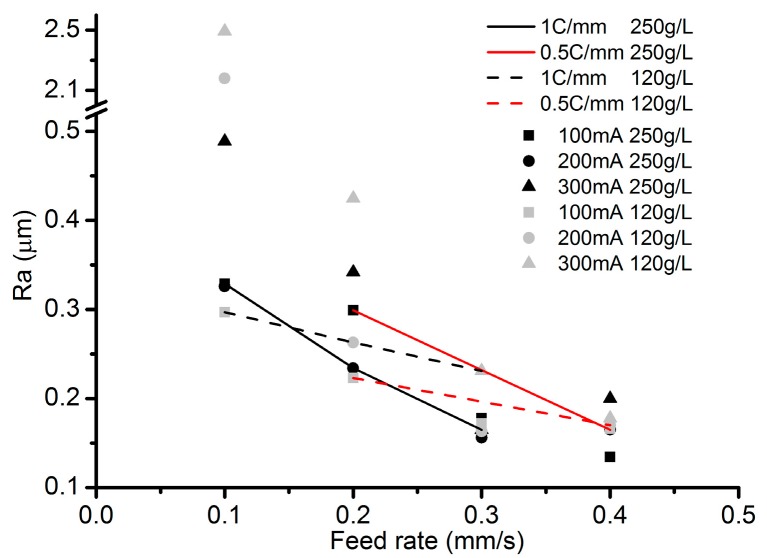
Roughness under different current values and concentrations.

**Figure 10:**
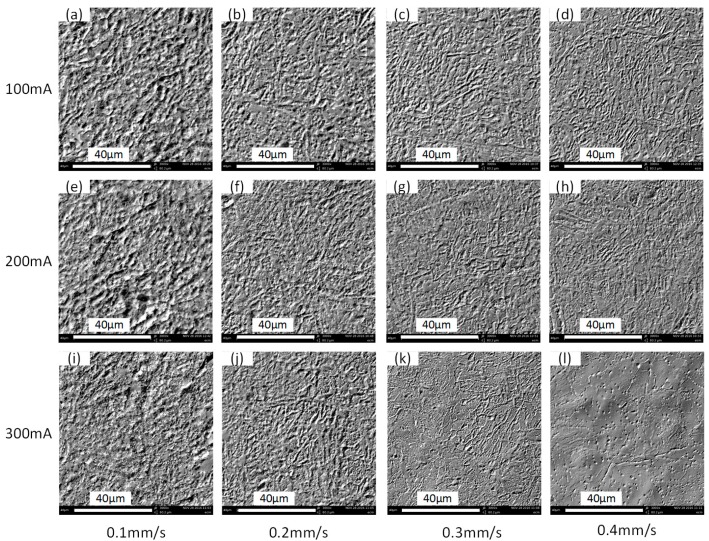
Microstructures of the channel bottom surface. (**a**) Current: 100 mA, feed rate: 0.1 mm/s; (**b**) current: 100 mA, feed rate: 0.2 mm/s; (**c**) current: 100 mA, feed rate: 0.3 mm/s; (**d**) current: 100 mA, feed rate: 0.4 mm/s; (**e**) current: 200 mA, feed rate: 0.1 mm/s; (**f**) current: 200 mA, feed rate: 0.2 mm/s; (**g**) current: 200 mA, feed rate: 0.3 mm/s; (**h**) current: 200 mA, feed rate: 0.4 mm/s; (**i**) current: 300 mA, feed rate: 0.1 mm/s; (**j**) current: 300 mA, feed rate: 0.2 mm/s; (**k**) current: 300 mA, feed rate: 0.3 mm/s; (**l**) current: 300 mA, feed rate: 0.4 mm/s.

Figure 9 indicates that, although the same ECPL can induce relatively uniform depth, the roughness declines along with the increase of the feed rate. Surface microstructures at the channel bottom as shown in Figure 10 also confirm this point when comparing Figure 10a,f,k. Rosenkranz et al. [27] has already pointed out, that during the ECM process, the iron surface is covered by a thin oxide layer, on top of which a polishing film of supersaturated iron nitrate forms. The thickness of film varies with the current density and the electrolyte flow rate, when other parameters do not change. With the increase of the applied current, the surfaces in each column of Figure 10, except the column with the feed rate of 0.1 mm/s, become smooth, which can be regarded as the result of the polishing film thickening. In each row of Figure 10, the surfaces become smooth along with the increase of the feed rate. This is because, when the current density near the droplet trail is much lower than the center area, a lower feed rate means a longer time of exposure to the low current density and this contributes to the thinning of the supersaturated film. Another noticeable problem in Figure 10 is that, although Figure 10l appears more smooth compared to other surfaces, its roughness is higher than that in Figure 10k. This may be explained by the time resolved model of the surface structure during ECM proposed by Rosenkranz et al. [27]. The proposed theory is that for very long current pluses or even direct current, the crystallographic orientation of the grains influence highly the electrochemical dissolution process and the roughness depends on the dissolution speed of different grains. From this point of view, the exposure to the low current density is possibly playing a positive role, because it can thin down the uneven polishing film induced by a large current density and flatten the machined surface. This might explain why, with the applied current of 300 mA, the feed rate of 0.3 mm/s produces a lower roughness compared to the feed rate of 0.4 mm/s.

### 3.3. Effects of Vacuum Gap (VG)

**Figure 11:**
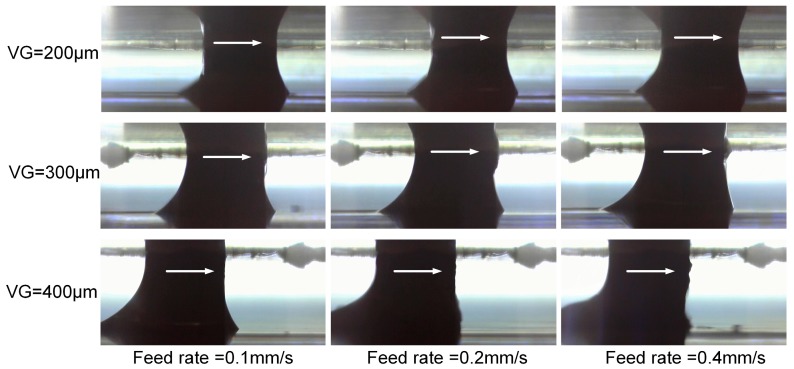
Droplet morphologies under different vacuum gap (VG).

The effects of VG have been also evaluated under a uniform air pressure. VG sizes of 200 µm, 300 µm and 400 µm have been, respectively, selected in the experiments to investigate its influence on the machining performance. The applied current setting was 200 mA and the IEG was set to 50 µm. In Figure 11, it can be noticed that, under the same feed rate, the trail of the droplet above the workpiece top surface becomes longer as the VG increases.

**Figure 12:**
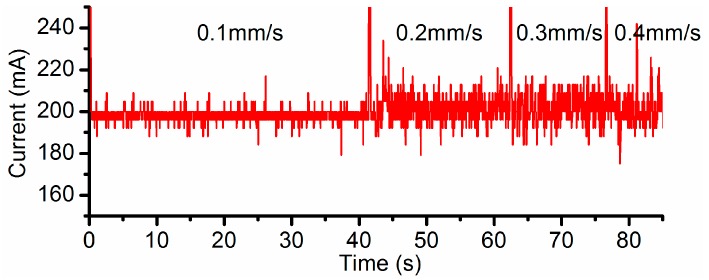
Current signal when setting 200 mA as the target (VG = 400 µm).

Figure 12 is the plot of the current signal under the VG of 400 µm. Compared to the situation with the feed rate of 0.1 mm/s, violent current fluctuations (~40 mA) have been detected around 200 mA in the three larger feed rates. This is a sign of instability in the flow field, which can also be confirmed by their corresponding droplet morphologies in Figure 11.

**Figure 13:**
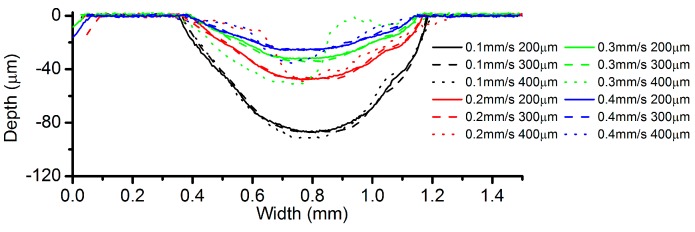
Cross-sectional profiles with different VGs (current = 200 mA).

**Figure 14:**
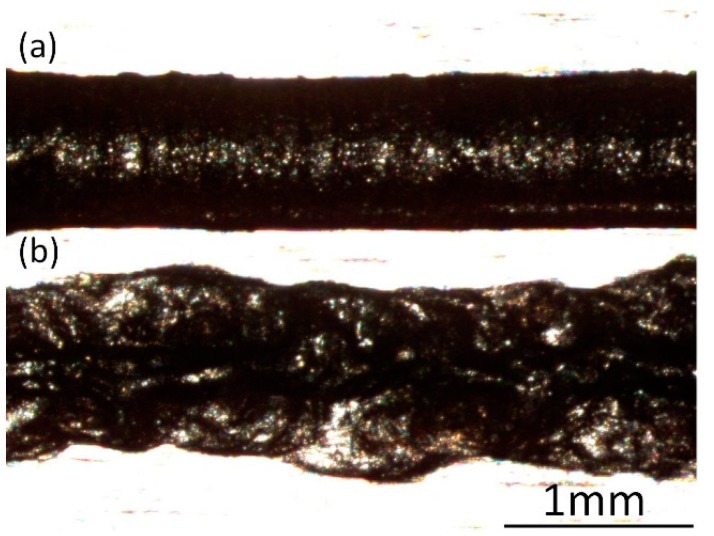
Channel morphologies under the VG of 400 µm at: (**a**) 0.1 mm/s; and (**b**) 0.2 mm/s.

Figure 13 portrays the cross-sectional profiles of the channels machined under different VGs and different feed rates. It can be noticed that when the VG is set to 400 µm, the cross-sectional profiles become irregular, apart from the case with a feed rate of 0.1 mm/s. Therefore, it can be assumed that there is a correlation between the fluctuation of current signals (Figure 12) and the profiles of the machined channels. Figure 14 exhibits clearly the morphology difference of the channels machined with feed rates of 0.1 mm/s and 0.2 mm/s. It can be seen that the channel with 0.2 mm/s has a very rough surface. Therefore, a VG of 400 µm can lead to an unstable machining performance, except for the cases with very small feed rate. It is noticeable that when the VG is set to 200 µm and 300 µm, there is no significant deviation between them, although some deviations exist in the morphologies of the moving droplets, as shown in the microscopic graphs (Figure 11).

Table 2 demonstrates the current efficiency when varying the VG. There is no definite discipline found on whether the VG affects the current efficiency.

**Figure 15:**
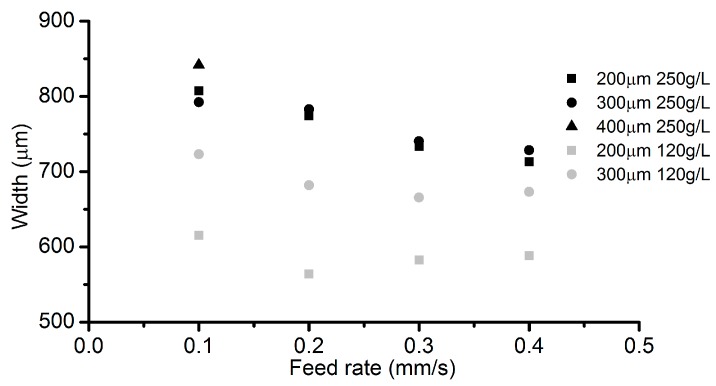
Channel width under different VGs.

**Figure 16:**
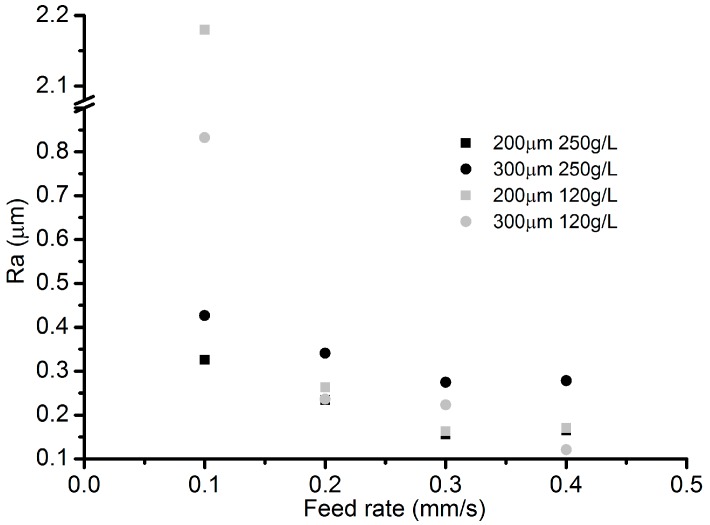
Roughness under different VGs.

The channel width under different VGs in Figure 15 indicates that a larger VG can lead to a larger channel width, especially in the case with low electrolyte concentration. Figure 16 reveals the roughness of the channel bottom surface under different VGs. With an electrolyte concentration of 250 g/L, a smaller VG (200 µm) results in a lower roughness. As for the electrolyte concentration of 120 g/L, the roughness trend shows an adverse effect.

**Figure 17:**
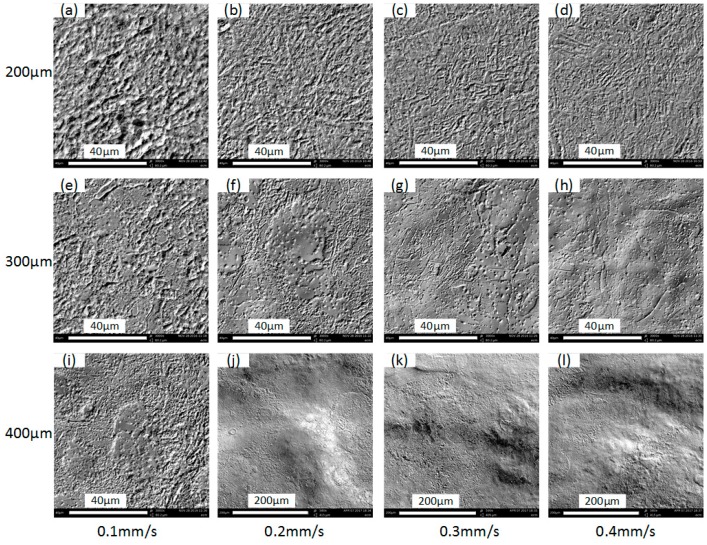
Surface microstructures under the current of 200 mA. (**a**) VG: 200 µm, feed rate: 0.1 mm/s; (**b**) VG: 200 µm, feed rate: 0.2 mm/s; (**c**) VG: 200 µm, feed rate: 0.3 mm/s; (**d**) VG: 200 µm, feed rate: 0.4 mm/s; (**e**) VG: 300 µm, feed rate: 0.1 mm/s; (**f**) VG: 300 µm, feed rate: 0.2 mm/s; (**g**) VG: 300 µm, feed rate: 0.3 mm/s; (**h**) VG: 300 µm, feed rate: 0.4 mm/s; (**i**) VG: 400 µm, feed rate: 0.1 mm/s; (**j**) VG: 400 µm, feed rate: 0.2 mm/s; (**k**) VG: 400 µm, feed rate: 0.3 mm/s; (**l**) VG: 400 µm, feed rate: 0.4 mm/s.

Figure 17 shows the surface microstructures under different VGs while the same electrolyte concentration is used (i.e., 250 g/L). Obvious heterogeneous microstructures distribute on the same surface when the VG is set to 300 µm and 400 µm, which spoils surface conformance and roughness. One possible reason for this phenomenon is that a larger VG could lead to a slower transport process and an increase in the concentration of the products, which further influences the formation of microstructures. From this point of view, it is better to apply as small as possible a VG to accelerate the update of the electrolyte under the condition of stabilizing the electrolyte droplet. Since a feed rate larger than 0.2 mm/s under the VG of 400 µm will result in irregular channel morphologies (as depicted in Figure 14), those corresponding surface microstructures are demonstrated in a larger view in Figure 17 to show the uneven surface.

### 3.4. Effects of Inter-Electrode Gap (IEG)

**Figure 18:**
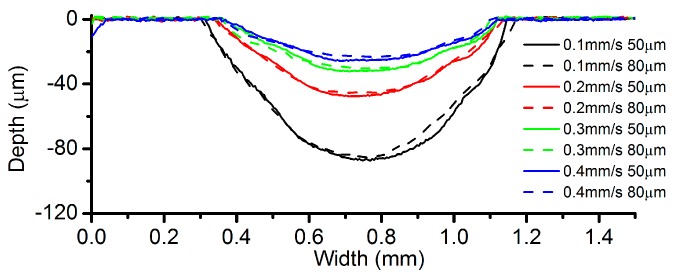
Cross-sectional profiles under different inter-electrode gaps (IEGs) (concentration: 250 g/L, current: 200 mA, VG: 200 µm).

**Figure 19:**
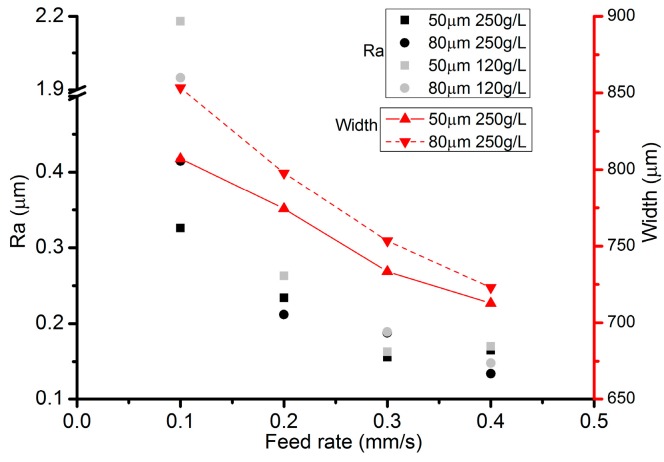
Channel roughness and width under different IEGs.

During the preliminary experiments, it has been identified that only an IEG below 100 µm was capable of stabilizing the electrolyte droplet. Thus, IEGs of 50 µm and 80 µm have been selected for further investigation. Figure 18 illustrates the cross-sectional profiles under different IEGs. It appears that the profile of the channels machined with the same feed rate has very small deviations in its maximum depth. Figure 19 demonstrates the variation of the roughness and width of the machined channels. The channels machined with a VG of 80 µm are wider than those with a VG of 50 µm, no matter what the feed rate is. This phenomenon can be explained by the fact that the applied voltage will be automatically elevated to maintain the specified current value when the IEG increases, which will in turn reduce the contact angle of the droplet and widen the contact area because of the electrowetting. As for the roughness, its relationship with the IEG still needs to be studied.

Table 3 shows the change of current efficiency with IEG. It can be noticed that the current efficiency in all cases with the IEG of 50 µm is larger than its counterpart at 80 µm.

### 3.5. Effects of Electrolyte Concentration

**Figure 20:**
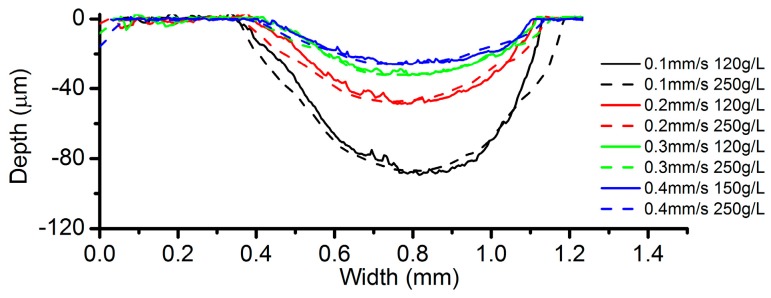
Cross-sectional profiles (current = 200 mA, VG = 200 µm).

**Figure 21:**
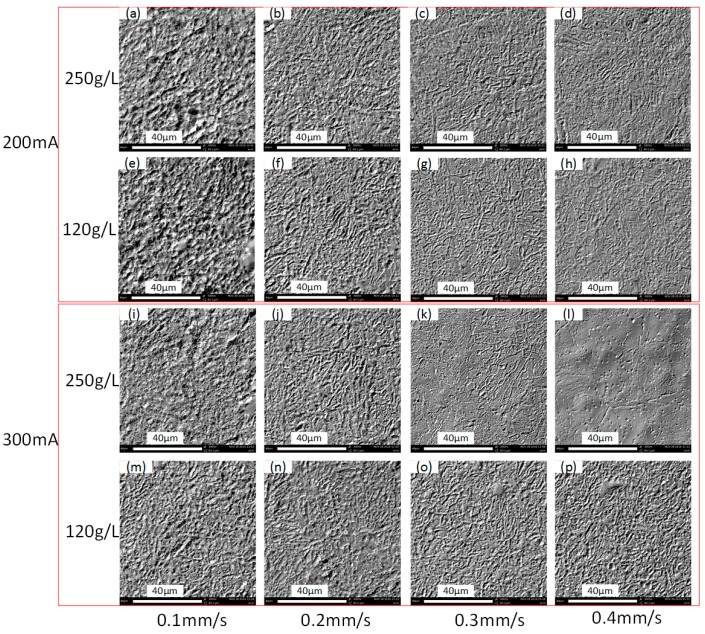
Microstructures of the channel bottom surface. (**a**) Current: 200 mA, electrolyte concentration: 250 g/L, feed rate: 0.1 mm/s; (**b**) current: 200 mA, electrolyte concentration: 250 g/L, feed rate: 0.2 mm/s; (**c**) current: 200 mA, electrolyte concentration: 250 g/L, feed rate: 0.3 mm/s; (**d**) current: 200 mA, electrolyte concentration: 250 g/L, feed rate: 0.4 mm/s; (**e**) current: 200 mA, electrolyte concentration: 120 g/L, feed rate: 0.1 mm/s; (**f**) current: 200 mA, electrolyte concentration: 120 g/L, feed rate: 0.2 mm/s; (**g**) current: 200 mA, electrolyte concentration: 120 g/L, feed rate: 0.3 mm/s; (**h**) current: 200 mA, electrolyte concentration: 120 g/L, feed rate: 0.4 mm/s; (**i**) current: 300 mA, electrolyte concentration: 250 g/L, feed rate: 0.1 mm/s; (**j**) current: 300 mA, electrolyte concentration: 250 g/L, feed rate: 0.2 mm/s; (**k**) current: 300 mA, electrolyte concentration: 250 g/L, feed rate: 0.3 mm/s; (**l**) current: 300 mA, electrolyte concentration: 250 g/L, feed rate: 0.4 mm/s; (**m**) current: 300 mA, electrolyte concentration: 120 g/L, feed rate: 0.1 mm/s; (**n**) current: 300 mA, electrolyte concentration: 120 g/L, feed rate: 0.2 mm/s; (**o**) current: 300 mA, electrolyte concentration: 120 g/L, feed rate: 0.3 mm/s; (**p**) current: 300 mA, electrolyte concentration: 120 g/L, feed rate: 0.4 mm/s.

The effects of the electrolyte concentration on the machining performance have already been partially illustrated in the sections above. Additional experiments have been conducted to investigate the surface microstructures derived under different electrolyte concentrations. The cross-sectional profiles in Figure 20 indicate that the electrolyte concentration has barely any effect on the maximum depth of machined channels. Figure 21 describes the surface microstructures obtained under different electrolyte concentrations. When the current is set to 200 mA, there is not much difference in the surface structures under the same feed rate. While with a current of 300 mA, there exist clear differences between images in Figure 21k,o as well as the images in Figure 21l,p. This discrepancy implies that the polishing film thickness depends heavily on the electrolyte concentration under higher current densities. This also corresponds to the case in Figure 9, in which a lower roughness is obtained under the electrolyte concentration of 120 g/L in comparison to the electrolyte concentration of 250 g/L. In summary, a lower electrolyte concentration is beneficial towards a smooth bottom surface because an uneven, thick and supersaturated layer formed under a high concentration electrolyte can deteriorate the roughness.

The calculated current efficiency in Table 4 shows that higher concentration contributes to a higher current efficiency compared with its lower-concentration counterpart.

### 3.6. Multiple Processing

**Figure 22:**
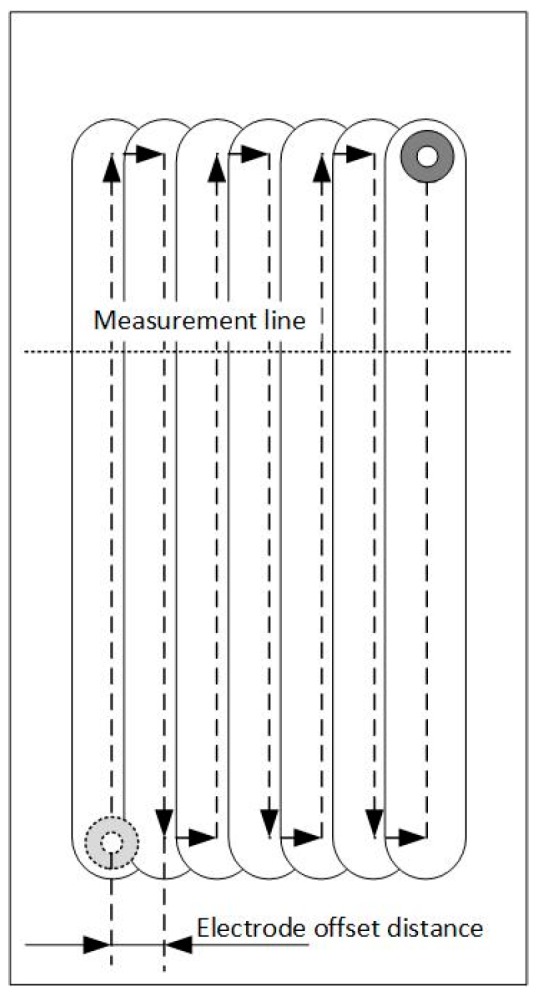
Electrode path.

Similar to using jet-ECM to generate complex surfaces by superimposed multi-dimensional motion [28] and to derive specified waviness through parameters adjustment [29], it is also possible to fabricate a pocket with the SMEFC by overlapping multiple process trajectories. In the experiments, the VG was set to 200 µm and the IEG was set to 50 µm as default unless otherwise specified. An overlapping of seven paths was adopted in a zigzag model as shown in Figure 22. The current settings and ECPL utilized in the experiments are described in Table 5. The feed rates were determined corresponding to the setting of ECPL. Three electrode offset distances (EOD), i.e., 200 µm, 300 µm and 400 µm, have been chosen for the experiments.

**Figure 23:**
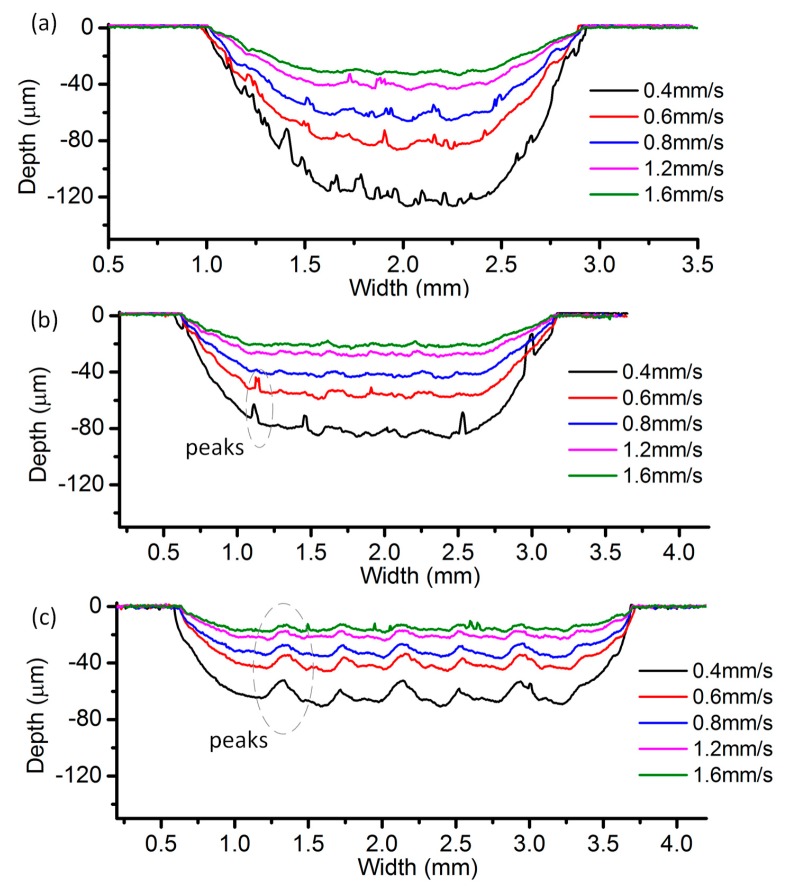
Cross-sectional profile with different electrode offset distances (EODs): (**a**) 200 µm; (**b**) 300 µm; and (**c**) 400 µm.

**Figure 24:**
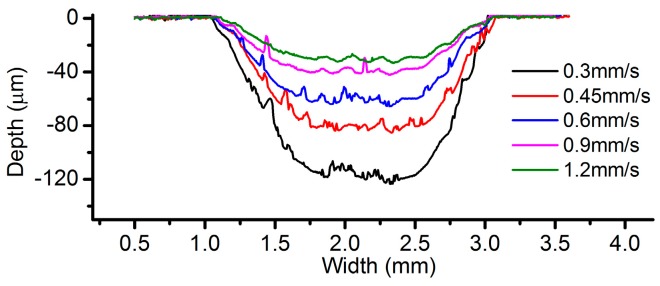
Cross-sectional profile with the current of 300 mA and EOD of 200 µm.

Figure 23 reveals the cross-sectional profiles of the cavities machined with different EODs with a current setting of 400 mA. It can be seen that the cavity depth increases as the EOD decreases, under the same feed rate. When the EOD is set to 400 µm (Figure 23c), there exist obvious periodic peaks on the bottom surface and the height of the peaks decrease along with the increase of the feed rate. Such kind of peaks cannot be observed in the profiles under the other two EODs. As shown in Figure 23a, when the feed rate is set at 1.6 mm/s, the bottom surface is relatively flat. However, in the case with four other feed rates, irregular spikes and valleys appear on the bottom surface. A possible reason for this phenomenon is that, with the same level of current, a small EOD will result in a relatively deep channel, which will in turn affect the flow field distribution of the neighboring electrolyte droplet. These effects tend to induce the electrolyte leakage and consequently deteriorate the bottom surface. A similar situation exists in Figure 24, which shows the cross-sectional profiles under the current of 300 mA and the EOD of 200 µm. When the EOD is set to 300 µm (Figure 23b), the bottom surface appears rather smooth except in the case feed rate of 0.4 mm/s.

**Figure 25:**
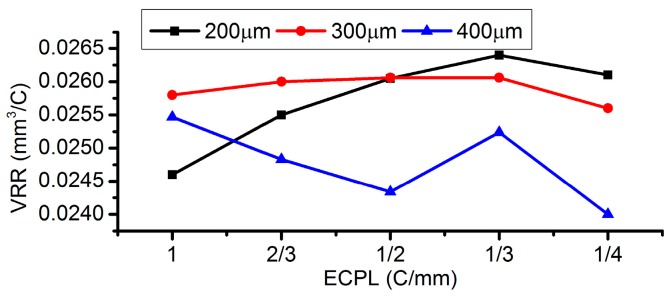
Volume removal rate (VRR) under different EODs.

The volume removal rate (VRR) under different EODs has also been calculated and drawn in Figure 25. It can be found that the MRR with an EOD of 300 µm remains at stable values around 0.0257 mm^3^/s, even if the ECPL changes from 1 C/mm to 1/4 C/mm. Considering both the flatness and the VRR stability, an EOD of 300 µm is a suitable value to be applied.

**Figure 26:**
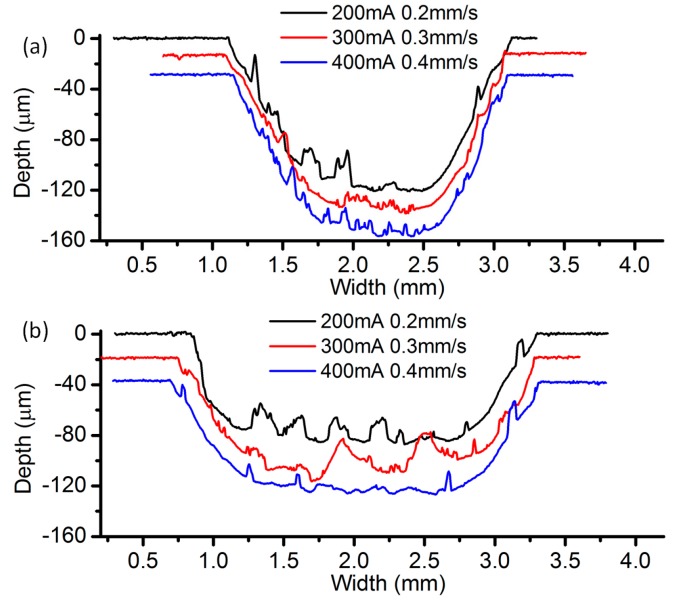
Cross-sectional profiles with different EODs: (**a**) 200 µm; (**b**) 300 µm.

**Figure 27:**
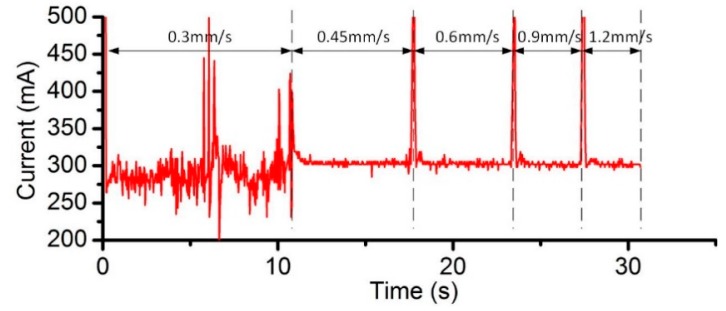
Current signal under 300 mA.

**Figure 28:**
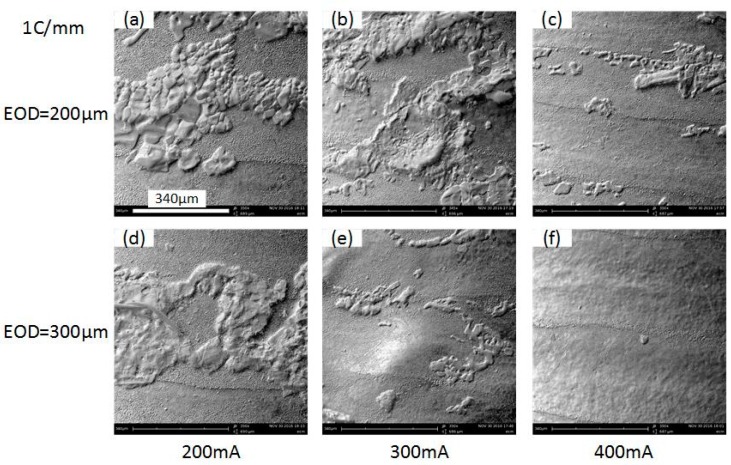
SEM pictures of the machined cavities. (**a**) EODs: 200 µm, current: 200 mA; (**b**) EODs: 200 µm, current: 300 mA; (**c**) EODs: 200 µm, current: 400 mA; (**d**) EODs: 300 µm, current: 200 mA; (**e**) EODs: 300 µm, current: 300 mA; (**f**) EODs: 300 µm, current: 400 mA.

The effect of the applied current has also been experimentally investigated, under the condition of the same ECPL. Figure 26 shows the comparison of the cross-sectional profiles under different current. It can be noticed that a better surface quality can be achieved under the current setting of 400 mA (Figure 26b). The case of 300 mA shows a rather rough profile and the corresponding current signal (Figure 27) fluctuates violently compared with other feed rates, which means the power supply needs more time to adjust the parameters to adapt the unstable flow field. When the EOD is set to 200 µm, rough cross-sectional profiles are formed no matter what the current setting is. To clarify this phenomenon, Figure 28 shows the bottom surface of the cavities machined under different EODs and the same ECPL of 1 C/mm. There are obvious residues on the surface of Figure 28a–c. Even with an EOD of 300 µm, residues still can be observed (in Figure 28d,e), but the cavity under the current setting of 400 mA shows a relatively smooth surface without any material residues as depicted in Figure 28f.

**Figure 29:**
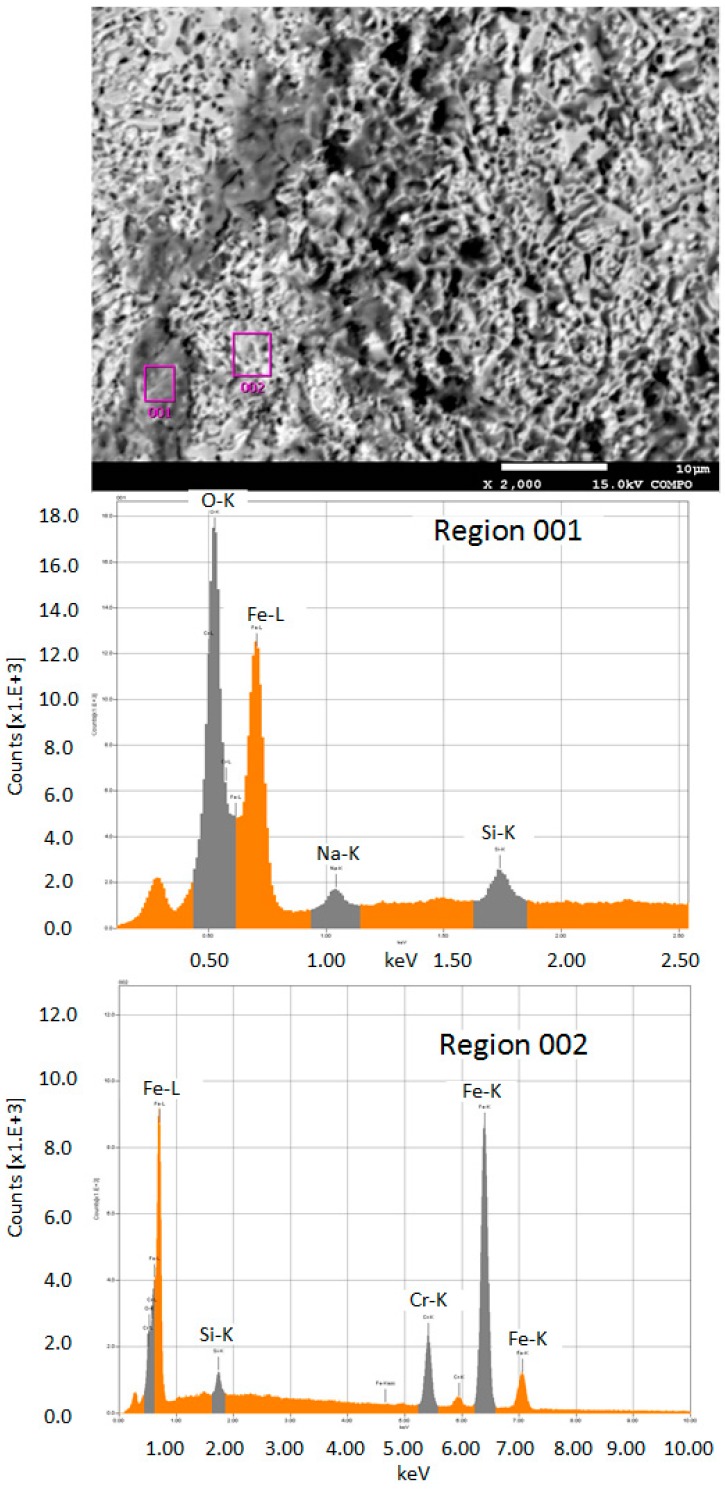
Energy-dispersive spectroscopy (EDS) analysis of the residues.

**Figure 30:**
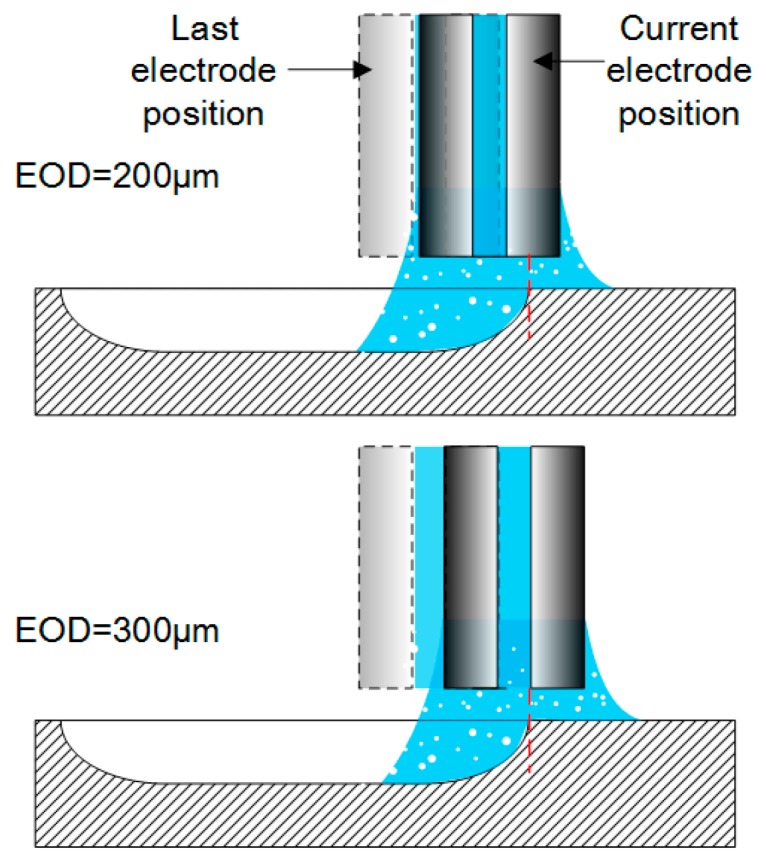
Schematic of different EODs.

In order to determine the residues composition, energy dispersive spectroscopy (EDS) analysis was conducted on a JXA-8530F (Jeol, Peabody, MA, UAS). Figure 29 shows the element spectrum and obvious peaks in Na element and O element were found in Region 001. It can be concluded that the residues on the machined surface is crystallization of NaNO_3_ salt. Figure 30 shows the schematic shape of the electrolyte droplet along the measurement line during the electrochemical dissolution. When EOD is 200 µm, there are more electrolytes in the cavity than the case with the EOD of 300 µm. It is more possible to generate electrolyte leakage when EOD is smaller. The reason why larger current induce less residues may be that, at the same level of ECPL, larger current means larger feed rate, which reduces the time of the electrolyte exposure to the fast airflow. This reduction inhibits the electrolyte crystallization possibility.

**Figure 31:**
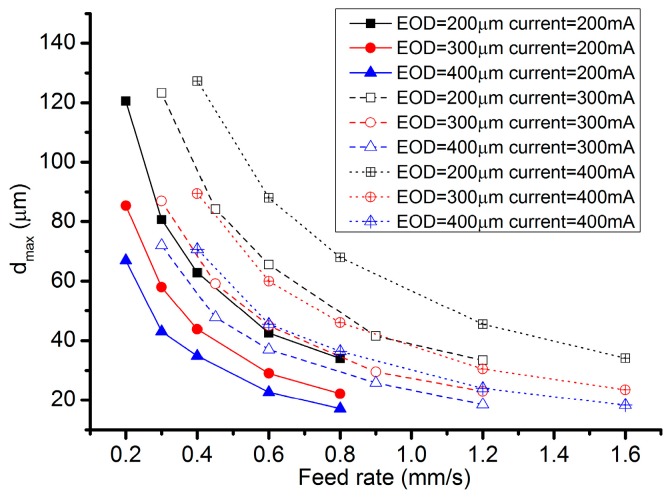
Maximum depth under different current values.

Figure 31 shows the maximum cavity depth (*d_max_*) obtained under different current settings. When the ECPL is kept the same, the *d_max_* always remains the same, although a higher current setting value will result in several microns larger than the *d_max_* derived by the lower current values.

Table 6 shows the quantitative fitting between the ECPL (*q*) and the *d_max_* at different combinations of the current and the EOD. It appears that the cavity depth has nearly a linear relationship with the ECPL variation, since the exponent of *q* is close to 1. With the increase of the current setting values, the maximum depth always grows in a small range.

## 4. Conclusions

1. An integrable scanning micro electrochemical flow cell (SMEFC) unit has been developed and utilized to fabricate mesoscale channels. The SMEFC can confine the electrolyte droplet just in the area of around 0.5 mm^2^ without leakage to the other non-processing region.
2. The roughness of the original workpiece surface affects the shape of the moving electrolyte droplet. Smooth surfaces tend to induce longer trials of the electrolyte droplet above the top of the workpiece surface.
3. Among all the geometrical parameters of the SMEFC configuration, the vacuum gap significantly affects the shape of the moving electrolyte droplet. A smaller vacuum gap tends to provide a better control of the electrolyte droplet, which contributes to the consistency of the surface microstructures. At the same level of the electric consumption per unit length, the channel depth maintain the same on the whole, but suitable combinations of the current densities and feed rates can generate better surface quality and roughness. The concentration of the electrolyte influences the formation of the supersaturated layer and further affects the roughness. A higher current density, a smaller inter-electrode gap and a higher electrolyte concentration improve the current efficiency.
4. As for the electrochemical milling by the SMEFC, the cavities machined with different electrode offset distances and different electric consumption per unit length have been compared. The electrode offset distance plays a significant role on the milling performance. Through the comparison of the cross-sectional profiles and SEM pictures, the electrode offset distance of 300 µm together with the current of 400 mA is a better combination for obtaining a relatively smooth bottom surface and a stable material removal rate. The removal depth can be adjusted by tuning the feed rate. A larger electrode offset distance and a higher current contribute to decrease of the residues of the electrolyte crystallization.
5. In future research, a linear power supply can replace the switching power supply for a better current holding capability. The electrode with the outer diameter of 0.3 mm will also be utilized to obtain smaller features. Multiple vision units will be installed at different angles to monitor and analyze the moving electrolyte droplet.

## Figures and Tables

**Table 1:** Experimental parameters.

Current (mA)	Feed Rate (mm/s)	ECPL (C/mm)	*S_exp_* (mm^2^)	*S_the_* (mm^2^)	η_1_	η_2_
100	0.1	1	0.0234	0.02327	100.6%	82.6%
200	0.2	1	0.0246	0.02327	105.7%	86.8%
300	0.3	1	0.0255	0.02327	109.6%	90.0%
100	0.2	0.5	0.0110	0.01165	94.4%	77.5%
200	0.4	0.5	0.0127	0.01165	109.0%	89.5%

**Table 2:** Current efficiency.

VG (µm)	Feed Rate (mm/s)	ECPL (C/mm)	*S_exp_* (mm^2^)	*S_the_* (mm^2^)	η_1_	η_2_
200	0.1	2	0.0498	0.0466	106.9%	87.8%
200	0.2	1	0.0246	0.0233	105.6%	86.7%
200	0.3	0.667	0.0164	0.0155	105.8%	86.9%
200	0.4	0.5	0.0127	0.01165	109.0%	89.5%
300	0.1	2	0.0499	0.0466	107.1%	87.9%
300	0.2	1	0.0246	0.0233	105.6%	86.7%
300	0.3	0.667	0.0169	0.0155	109.0%	89.5%
300	0.4	0.5	0.0122	0.01165	104.7%	85.9%
400	0.1	2	0.0499	0.0466	107.1%	87.9%

**Table 3:** Current efficiency.

IEG (µm)	Feed Rate (mm/s)	ECPL (C/mm)	*S_exp_* (mm^2^)	*S_the_* (mm^2^)	η_1_	η_2_
50	0.1	2	0.0498	0.0466	106.9%	87.8%
80	0.1	2	0.0477	0.0466	102.4%	84.1%
50	0.2	1	0.0246	0.0233	105.6%	86.7%
80	0.2	1	0.0245	0.0233	105.6%	86.7%
50	0.3	0.667	0.0164	0.0155	105.8%	86.9%
80	0.3	0.667	0.0161	0.0155	103.9%	85.3%
50	0.4	0.5	0.0127	0.01165	109.0%	89.5%
80	0.4	0.5	0.0120	0.01165	103.0%	84.6%

**Table 4:** Current efficiency.

Concentration (g/L)	Feed Rate (mm/s)	ECPL (C/mm)	*S_exp_* (mm^2^)	*S_the_* (mm^2^)	η_1_	η_2_
120	0.1	2	0.0473	0.0466	101.5%	83.3%
250	0.1	2	0.0498	0.0466	106.9%	87.8%
120	0.2	1	0.0242	0.0233	103.9%	85.3%
250	0.2	1	0.0246	0.0233	105.6%	86.7%
120	0.3	0.667	0.0152	0.0155	98.1%	80.5%
250	0.3	0.667	0.0164	0.0155	105.8%	86.7%
120	0.4	0.5	0.0116	0.01165	99.6%	81.8%
250	0.4	0.5	0.0127	0.01165	109.0%	89.5%

**Table 5:** Feed rate (mm/s) used in the experiments.

	ECPL (C/mm)	1	2/3	1/2	1/3	1/4
Current (mA)						
200		0.2	0.3	0.4	0.6	0.8
300		0.3	0.45	0.6	0.9	1.2
400		0.4	0.6	0.8	1.2	1.6

**Table 6:** *d_max_* (µm) fitting equations.

	EOD (µm)	200	300	400
Current (mA)				
200		$120\times q^{0.9365}$	$85.49\times q^{0.9714}$	$66.5\times q^{0.9821}$
300		$123.7\times q^{0.9516}$	$87.17\times q^{0.9657}$	$71.72\times q^{0.9607}$
400		$127.8\times q^{0.9326}$	$89.41\times q^{0.9702}$	$70.15\times q^{0.9807}$

## References

[1] Lee S.-J., Lee C.-Y., Yang K.-T., Kuan F.-H., Lai P.-H. (2008). Simulation and fabrication of micro-scaled flow channels for metallic bipolar plates by the electrochemical micro-machining process. J. Power Sources.

[2] Hung J.-C., Chang C.-H., Chiu K.-C., Lee S.-J. (2013). Simulation-based fabrication of micro-helical grooves in a hydrodynamic thrust bearing by using ecmm. Int. J. Adv. Manuf. Technol..

[3] Liu G.X., Zhang Y.J., Jiang S.Z., Liu J.W., Gyimah G.K., Luo H.P. (2016). Investigation of pulse electrochemical sawing machining of micro-inner annular groove on metallic tube. Int. J. Mach. Tools Manuf..

[4] Ryu S.H. (2015). Eco-friendly ecm in citric acid electrolyte with microwire and microfoil electrodes. Int. J. Precis. Eng. Manuf..

[5] Jia L., Xiaochen J., Di Z. (2016). Electrochemical machining of multiple slots with low-frequency tool vibrations. Procedia CIRP.

[6] Liu G., Zhang Y., Deng Y., Wei H., Zhou C., Liu J., Luo H. (2017). The tool design and experiments on pulse electrochemical machining of micro channel arrays on metallic bipolar plate using multifunctional cathode. Int. J. Adv. Manuf. Technol..

[7] Natsu W., Ikeda T., Kunieda M. (2007). Generating complicated surface with electrolyte jet machining. Precis. Eng..

[8] Hackert M., Meichsner G., Schubert A. Generating micro geometries with air assisted jet electrochemical machining. Proceedings of the Euspen 10th Anniversary International Conference.

[9] Kai S., Sai H., Kunieda M., Izumi H. (2012). Study on electrolyte jet cutting. Procedia CIRP.

[10] Kunieda M., Mizugai K., Watanabe S., Shibuya N., Iwamoto N. (2011). Electrochemical micromachining using flat electrolyte jet. CIRP Ann. Manuf. Technol..

[11] Hackert-Oschätzchen M., Meichsner G., Zinecker M., Martin A., Schubert A. (2012). Micro machining with continuous electrolytic free jet. Precis. Eng..

[12] Ghoshal B., Bhattacharyya B. (2015). Investigation on profile of microchannel generated by electrochemical micromachining. J. Mater. Process. Technol..

[13] Ghoshal B., Bhattacharyya B. (2016). Electrochemical micromachining of microchannel using optimum scan feed rate. J. Manuf. Process..

[14] Kim B.H., Ryu S.H., Choi D.K., Chu C.N. (2005). Micro electrochemical milling. J. Micromech. Microeng..

[15] Zhang Z., Zhu D., Qu N., Wang M. (2007). Theoretical and experimental investigation on electrochemical micromachining. Microsyst. Technol..

[16] Shin H.S., Kim B.H., Chu C.N. (2008). Analysis of the side gap resulting from micro electrochemical machining with a tungsten wire and ultrashort voltage pulses. J. Micromech. Microeng..

[17] Wang S., Zhu D., Zeng Y., Liu Y. (2011). Micro wire electrode electrochemical cutting with low frequency and small amplitude tool vibration. Int. J. Adv. Manuf. Technol..

[18] Liu Z., Nouraei H., Spelt J.K., Papini M. (2015). Electrochemical slurry jet micro-machining of tungsten carbide with a sodium chloride solution. Precis. Eng..

[19] Yuan L., Xu J., Zhao J., Zhang H. (2012). Research on hybrid process of laser drilling with jet electrochemical machining. J. Manuf. Sci. Eng..

[20] Zhang Z., Cai M., Feng Q., Zeng Y. (2014). Comparison of different laser-assisted electrochemical methods based on surface morphology characteristics. Int. J. Adv. Manuf. Technol..

[21] Sakairi M., Sato F., Gotou Y., Fushimi K., Kikuchi T., Takahashi H. (2008). Development of a novel microstructure fabrication method with co-axial dual capillary solution flow type droplet cells and electrochemical deposition. Electrochim. Acta.

[22] Drensler S., Milenkovic S., Hassel A.W. (2014). Microvials with tungsten nanowire arrays. J. Solid State Electrochem..

[23] Hu J.-F., Kuo C.-L. (2007). Study on Micro Electrochemical Machining Using Coaxial for Gushing and Sucking Method. Master’s Thesis.

[24] Kuo K.-Y., Wu K.-L., Yang C.-K., Yan B.-H. (2013). Wire electrochemical discharge machining (WECDM) of quartz glass with titrated electrolyte flow. Int. J. Mach. Tools Manuf..

[25] Leroy P., Lassin A., Azaroual M., André L. (2010). Predicting the surface tension of aqueous 1:1 electrolyte solutions at high salinity. Geochim. Cosmochim. Acta.

[26] Deconinck D., Hoogsteen W., Deconinck J. (2013). A temperature dependent multi-ion model for time accurate numerical simulation of the electrochemical machining process. Part III: Experimental validation. Electrochim. Acta.

[27] Rosenkranz C., Lohrengel M., Schultze J. (2005). The surface structure during pulsed ecm of iron in NaNO_3_. Electrochim. Acta.

[28] Schubert A., Hackert-Oschätzchen M., Martin A., Winkler S., Kuhn D., Meichsner G., Zeidler H., Edelmann J. (2016). Generation of complex surfaces by superimposed multi-dimensional motion in electrochemical machining. Procedia CIRP.

[29] Martin A., Eckart C., Lehnert N., Hackert-Oschätzchen M., Schubert A. (2016). Generation of defined surface waviness on tungsten carbide by jet electrochemical machining with pulsed current. Procedia CIRP.

